# Notes and News

**Published:** 1902-02-22

**Authors:** 


### NOTES AND NEWS.

### HOSPITAL MEETINGS.

Queen Charlotte's Hospital.—Annual Meeting at the Hospital, Monday, February 24th, at 3 p.m.

Tottenham Hospital.—Annual Meeting at the Hospital, Tuesday, February 25th, at 5.15 p.m.

Cancer Hospital.—Annual Meeting at the Hospital, Wednesday, February 26th, at 4 p.m.

King's College Hospital.—Annual Meeting at the Hospital, Thursday, February 27th, at 4 p.m.

Middlesex Hospital.—Annual Meeting at the Hospital, Thursday, February 27th, at 12 noon.

Great Northern Central Hospital.— Annual Meeting at the Hospital, Friday, February 28th, at 5 p.m.

Seamen's Hospital —Annual Meeting at the Institute of Chartered Accountants, Moorgate Place, Friday, February 28th, at 3.30 p.m.

National Orthopædic Hospital.—Annual Meeting at the Hospital, Friday, February 28th, at 4 p.m.

Royal London Ophthalmic Hospital.—Annual Meeting, Thursday, February 27th, at 4 p.m.

Sir Thomas Brocklebank has presented £1,000 to the Liverpool Hospital for cancer and diseases of the skin.

Sir Francis Lovell's mission to India in connection with the London School of Tropical Diseases is proving very successful in creating interest and securing support.

Surgeon-Captain T. Crean is to receive the Victoria Cross for his gallant behaviour in tending the wounded at Tygerskloof in December last under fire, he himself being wounded at the time.

The second Lettsonian Lecture of the Medical Society of London will be delivered by Mr. A. Pearce Gould, on March 3rd. The subject will be "Obliterative Arteritis." The third lecture will be given on March 17th, on "Thrombosis."

Owing to the generosity of Mr. and Mrs. John Dyer, a children's ward has been opened at the Swansea Hospital. The opening ceremony was performed in a novel and practical way by Mrs. Dyer, who carried the first small patient into the ward. Previously the children had been treated with adults at Swansea, and this plan has been found, as elsewhere, to have several drawbacks. The ward now opened was an unused one, and is now excellently furnished by the donors. Some of the windows are of stained glass, containing illustrations of nursery tales.

The French Society of the History of Medicine has been established with Professor Raphael Blanchard as its president.

An International Tuberculosis Bureau has been formed. Amongst its honorary members are already representatives of a large number of nationalities. Sir William Broadbent and Sir Hermann Weber represent Great Britain.

The Militia Medical Staff Corps and the Volunteer Medical Staff Corps are, in consideration of their services in South Africa, both to receive the title of Royal, and the officers will drop the prefix of surgeon to their military title throughout all grades.

The seventieth annual meeting of the British Medical Association will be held at Manchester, commencing on July 29th. The President-elect is Walter Whitehead, F.R.C.S. Edin., F.R.S.E., Consulting Surgeon Manchester Royal Infirmary. An Address in Medicine will be delivered by Sir Thomas Barlow, Bart., K.C.V.O., M.D., and one on Obstetrics by William Japp Sinclair, M.D.

The attention attracted to the subject of small-pox at the present time seems to be universal. In New York the City Board of Health have increased their vaccinating staff by one hundred. Each borough of the city is to have a disinfecting plant, and an isolation hospital is to be built on Staten Island.

The present prevalence of the plague in India is very disappointing. The numbers are considerably higher than those of last year. In the Bombay Presidency this is especially so, and in the Punjab it has gained a far more serious hold. The total mortality throughout India during one week has recently amounted to 10,000. The Sultan of Turkey has just issued orders for the establishment of a bacteriological laboratory for the study of the plague in an isolated spot on the Bosphorus.

The Association Internationale de la Presse Medicale proposes to hold a meeting at Monte Carlo on April 7th. This meeting will be composed of national delegates nominated by the existing medical press associations in each country. Among other matters to be discussed will be a project for the establishment of a permanent international bureau which shall receive résumés of medical works from the entire world, edited by the authors, in order that they may be distributed among the journals forming part of the International Association.

During the past year there has been a great deal of small-pox in the United States. The Surgeon-General reports that during 1901 this disease occurred in every State and Territory of the Union, with the exception of Arizona, from which no report had been received. The number of cases reported during the year had amounted to 38,506, and the deaths to 689. Thus widespread as the disease has been, it must on the whole have assumed a very mild form, the mortality only amounting to 1·79 per cent. of the cases; very different from the high fatality which prevails among the cases now occurring in London.

